# Statistical Analysis of Bistatic Radar Ground Clutter for Different German Rural Environments

**DOI:** 10.3390/s20113311

**Published:** 2020-06-10

**Authors:** Michael Kohler, Daniel W. O’Hagan, Matthias Weiss, David Wegner, Josef Worms, Oliver Bringmann

**Affiliations:** 1 Department of Passive Radar and Anti-Jamming Techniques, Fraunhofer Institute for High Frequency Physics and Radar Techniques FHR, 53343 Wachtberg, Germany; daniel.ohagan@fhr.fraunhofer.de (D.W.O.); matthias.weiss@fhr.fraunhofer.de (M.W.); david.wegner@fhr.fraunhofer.de (D.W.); josef.worms@fhr.fraunhofer.de (J.W.); 2 Department of Computer Science, University of Tübingen, 72076 Tübingen, Germany; oliver.bringmann@uni-tuebingen.de

**Keywords:** bistatic radar, passive bistatic radar, bistatic clutter, rural terrain clutter, rural ground clutter

## Abstract

This article presents the statistical analysis of bistatic radar rural ground clutter for different terrain types under low grazing angles. Compared to most state-of-the-art analysis, we present country-specific clutter analysis for subgroups of rural environments rather than for the rural environment as a whole. Therefore, the rural environment analysis is divided into four dominant subgroup terrain types, namely fields with low vegetation, fields with high vegetation, plantations of small trees and forest environments representing a typical rural German environment. We will present the results for both the summer and the winter vegetation. Therefore, bistatic measurement campaigns have been carried out during the summer 2019 and the winter of 2019/20 in the aforementioned four different rural terrain types. The measurements were performed in the radar relevant X-band at a center frequency of 8.85 GHz and over a bandwidth of 100 MHz according to available transmit permission. The distinction of the rural terrain into different subgroups enables a more precise and accurate clutter analysis and modeling of the statistical properties as will be shown in the presented results. The statistical properties are derived from the calculated clutter amplitudes probability density functions and corresponding cumulative distribution functions for each of the four terrain types and the corresponding season. The data basis for the clutter analysis are the processed range-Doppler maps from the bistatic radar measurements. According to the authors’ current knowledge, a similar investigation based on real bistatic radar measurement data with the division into terrain subgroups has not yet been carried out and published for a German rural environment.

## 1. Introduction

Bistatic and passive bistatic radar are currently the subjects of high research interest where the name passive bistatic radar is given to a bistatic radar that uses illuminators of opportunity (PCL) e.g., broadcast or communications transmitters rather than a cooperative radar transmitter [1,2,3,4,5,6]. In a bistatic radar setup, the transmitter and receiver are spatially separated. The bistatic radar receiver usually receives the desired target echo, the direct signal from the transmitter and the unwanted signal reflections caused by e.g., obstacles within the illuminated scene. In the terminology of radar, these unwanted signal reflections and receptions are commonly known as clutter.

In this article, the focus is on bistatic rural ground clutter which is meant to be clutter caused by rural environments in ground-based bistatic or passive bistatic radar applications. The performance of a ground-based bistatic radar in the context of target detection is particularly dependent on the characteristics and statistical parameters of this clutter. In general, rural ground clutter can be fluctuating or non-fluctuating. Accordingly, clutter can propagate both in bistatic range and in Doppler dimension decreasing target detection and tracking performance. Due to movements of the illuminated scene e.g., movements of trees due to the wind the clutter spreads also in the Doppler dimension. Depending on the clutter strength, a target echo can be masked leading to a decrease in detection performance. Also, the clutter sidelobes in the bistatic range and Doppler domain can cause a target echo masking. Therefore, a sufficient clutter suppression is desired which involves a-priori knowledge of the clutter statistics. The a-priori knowledge of the clutter characteristics can also be exploited to adaptive illumination and waveform design. The adaptation and optimization of the radar waveform to a target in a given environment with respect to a certain criterion such as signal-to-interference-plus-noise ratio (SNIR) requires knowledge of the clutter statistics [7,8,9] in order to optimize the waveform parameters. The emphasis of this article is the investigation of bistatic ground clutter for the X-band of German rural environments under low grazing angles. It should be mentioned that in clutter analysis the grazing angle is defined as the elevation angle between the antenna boresight and the clutter surface [10]. The backscattering properties of the clutter source depend, among other things, on this angle. For ground-based bistatic applications, as treated in this article, small grazing angles are of interest. There are already numerous publications about rural ground clutter for the X-band frequency range which will be discussed in the following. Fundamental research on the influence of several composite North-American rural terrain types including, among others, farmland, forests, lakes or mountains under low grazing angles has been addressed in several publications by J. B. Billingsley [11,12,13]. The authors present clutter models including the histograms, momentum analysis and goodness-of-fit testing for various terrain types e.g., scrub desert, rangeland, cropland vegetation and forests according to the country-specific vegetation where the measurements have been carried out. Further statistical clutter analysis for windblown foliage also considering forest environments using spectral measurements has been presented in [14,15,16,17]. The authors present amplitude models describing the clutter distribution for different types of terrain, terrain-dependent damping factors and analytical descriptions of Doppler models for different wind speeds. Gaussian, power-law and double-exponential spectral models are used to describe the clutter spread in the Doppler dimension for the windblown terrains. The influence of the seasons on the clutter properties has also been investigated. For this purpose, measurements were carried out for different types of terrain in different seasons. Further statistical clutter parameters and probability density functions on the clutter influence for different specific forest components e.g., canopies or leaves and branches of the trees are presented in [18]. It was shown that the amplitude distribution of the clutter values for leaves smaller than the incident wavelength can be described by a Rayleigh distribution. For fouling in the order of magnitude of the incident wavelength, the amplitudes are distributed according to a Gamma distribution. The authors also describe the geometric simulation of the different plant characteristics. For further composite terrains also including farmland and other country-specific rural terrain types several statistical clutter parameters based on amplitude statistics are presented in [19]. The authors present a derived Poisson distribution to model the amplitude distribution with empirically adapted parameters to describe composite rural environments for high as well as low grazing angles. A key parameter is the clutter type, where the authors distinguish between weak, medium and strong backscatterers. Statistical clutter models for spatial amplitude statistics of rural ground-based clutter are presented in [20]. Terrain-specific parameters of a Weibull distribution describing the clutter amplitudes for low grazing angles are presented, whereby a distinction is made between the terrain types general rural, open farmland, continuous forest and meadows. The distributions show a clear separation of meadows from the other types of terrain. Spectral component analysis of ground clutter for agricultural land, farmland and forest environments are described in [21]. One of the main statements of the work is the composition of the Doppler models. A distinction is made between a coherent and a diffuse component. According to the authors, the coherent component is mainly formed by unbalanced objects such as tree trunks, whereas the moving objects form the diffuse component. Grass- and soil-based clutter has been investigated in [22]. The authors describe the amplitude distributions with a Weibull distribution. In addition, the shift of the distribution with increasing grazing angle towards lower attenuation values was worked out. The influence of an illumination with a low grazing angle range for Indian rural terrain on the statistical clutter properties is presented in [23]. A comprehensive database considering different clutter density functions can be found in [24] also for snow covered terrain. Another example for country-specific analysis are Doppler spectrum-based clutter models for South African urban terrain which can be found in [25]. Amplitude-based clutter models specific for different Saudi Arabian rural terrain are presented in [26] for low grazing angles. Clutter statistics for cultivated land, wooded hills and forest also for different seasons are presented in [27]. Further land clutter models and the corresponding probability density functions for bistatic applications are presented in [28]. The analysis of clutter caused by palm trees considering their unique structure has been addressed in [29].

In this article, we present a low grazing angle statistical clutter analysis for German rural environments. The statistical parameters have been derived from real measurement data. Therefore, ground-based bistatic radar measurements have been carried out in 2019 and 2020. The measurements have been performed in the radar relevant X-band and so this article presents statistical clutter models according to this frequency range. To investigate the seasonal influence on the results, the measurements were carried out both in summer and winter. According to the rural environments prevailing in Germany, the measurements took place in four dominant rural terrain subgroups. These four rural terrain subgroups are listed below:

- Fields with low vegetation
- Fields with high vegetation
- Plantations with small trees
- Forest environments

Fields with low vegetation stand for areas which mainly consist of meadows, farmland or other agricultural areas with low vegetation. Fields with high vegetation are correspondingly the group of agricultural areas with high vegetation such as corn, wheat or grain fields. The third type of terrain is made up of plantations of small trees such as fruit tree plantations, vineyards or other arrangements of smaller trees. Forests form the fourth subgroup. Together, these four groups largely represent a typical German rural environment. According to the knowledge of the authors, such measurement campaigns to collect data of low grazing angle German rural terrain clutter is the first of its kind at the time of writing this article. A ground-based bistatic measurement setup has therefore been developed to carry out the measurements. A dual-channel receiver was used as bistatic radar receiver for the measurements. The direct signal of the transmitter is delivered via one receiving channel and the clutter echoes of the corresponding terrain under test are received with the second channel. A cooperative X-band transmitter was used to illuminate the terrain during each measurement. The sampled data was then processed and an amplitude-based statistical clutter analysis was performed on the resulting range-Doppler map data. In Section 2 the measurement campaigns, the developed bistatic radar setup as well as the developed signal processing approach for the two receiving channels are presented. Also in Section 2 the statistical parameters that will be used in this article to characterize the clutter are presented. In Section 3 the results of the clutter amplitude analysis for the different types of terrain and the different seasons are shown. A discussion of the results achieved is given in Section 4. A conclusion and outlook on our future work is given in Section 5.

## 2. Materials and Methods

### 2.1. Measurement Campaigns

Several measurement campaigns in different seasons have been carried out to analyze the bistatic radar rural ground clutter of German rural environments for a low grazing angle. Therefore, the rural environment has been divided into four dominant groups, namely fields with low vegetation, fields with high vegetation, plantations of small trees and forest environments. These are mainly mixed forests consisting of spruce, oak, beech and pine trees.

**Figure 1:**
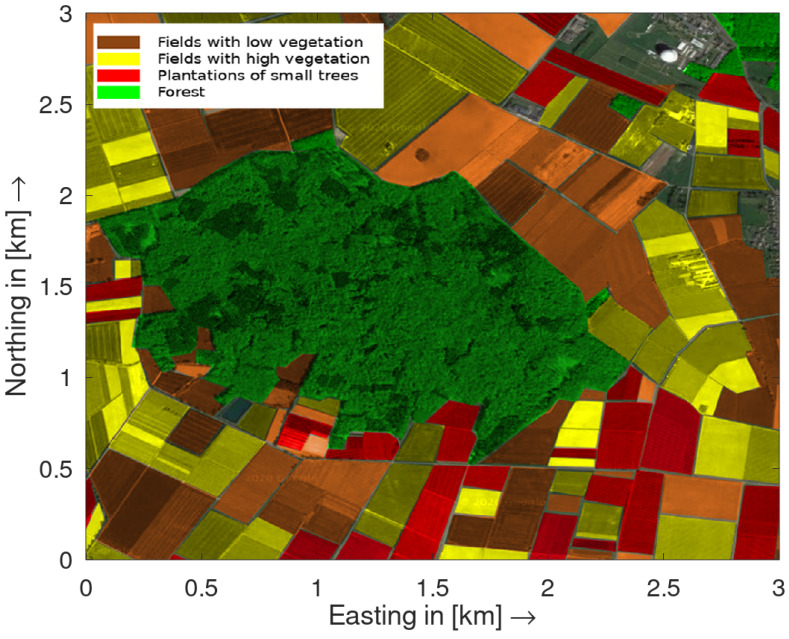
A typical German rural terrain in the Eifel region of western Germany with the colored different terrain groups specified for the bistatic measurements and clutter analysis.

Such a division allows a more specific study of the statistical clutter properties compared to a holistic view of the rural terrain. Figure 1 shows an example of a typical German rural environment with the highlighted aforementioned subgroups. The terrain characteristics shown are representative for the terrain in which the measurements were carried out in the Eifel region in western Germany around the Fraunhofer Institute FHR also shown in the top right corner. According to Figure 1 the covered landmass is 32% for fields with low vegetation, 23% for fields with high vegetation, 11% for plantations of small trees and 29% for forests. Analogous to the official data from the German Federal Statistical Office, the proportion of forest areas is 29.8% [30]. The share of agricultural land is 50.08% in total. This includes the groups fields with low vegetation, high vegetation and plantations of small trees. The official numbers for the fields with low vegetation show a share of 70.5%, 27.2% for the fields with high vegetation and 1.2% for the plantations of small trees [31,32]. These data represent an average value for the whole land mass of Germany. Nevertheless, fields with low vegetation make up the largest part, followed by fields with high vegetation and plantations of small trees. This order can also be validated with the official statistical data and thus correlates with the data presented here. These ratios already show that a holistic view for such a rural environment as a homogeneous land mass is not sufficient. A bistatic radar setup was developed for the clutter measurements in the different terrains. For each type of terrain, bistatic measurements were carried out at four independent positions in the summer of 2019. In addition, the measurements were repeated in the winter 2019/2020 for all terrain types except for the fields with high vegetation since these are not available at this time of year. The transmitter and receiver were placed around the terrain under test with an angle of 45° between transmit and reference channel antenna boresight. According to the terrain dimensions, the baselines varied between 300 m and 1000 m. During each measurement the grazing angle was below 5°. The center frequency was set to 8.85 GHz with vertically polarized transmission. The radar waveform used was a linear frequency modulated pulse with a bandwidth of 100 MHz and a transmit power of 30 dBm according to the transmission regulations. The pulse on-time was set 15 μs and the off-time to 5 μs.

### 2.2. The Bistatic Radar Setup

To be able to collect data for a statistical bistatic ground clutter analysis, a measurement setup was developed. The setup consists of a transmitter and a bistatic dual-channel receiver. Since a direct decoupling of the signal on the transmitter side is not possible, one receiving channel is used to receive the direct signal and the second channel to receive the clutter echoes of the terrain under test for further processing. The special feature of this setup is the external synchronization of the transmitter and receiver via GPS disciplined rubidium clocks. Using an external reference signal generation, enables phase coherent measurements resulting in a higher signal-to-noise ratio. In the following, the schematic diagrams of the transmitter and receiver are presented.

#### 2.2.1. The Dual-Channel Bistatic Receiver

**Figure 2:**
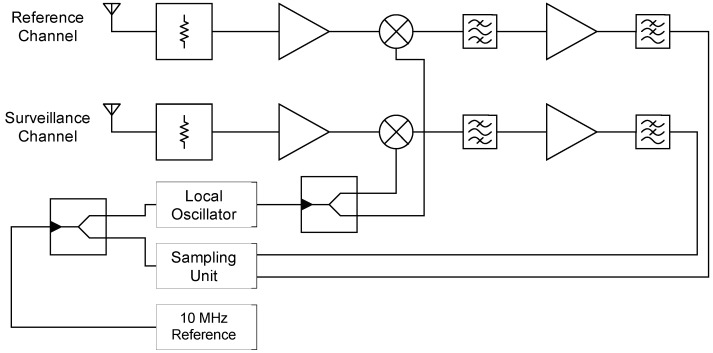
The dual-channel receiver developed for the bistatic radar measurements with a reference channel for the reception of the direct signal and a surveillance channel for the clutter reception.

In Figure 2 the structural diagram of the dual-channel bistatic radar receiver is shown. The receiver is designed for the X-band and can receive signals with a bandwidth of 100 MHz using a tunable local oscillator. Both receiving channels have an identical front-end design. Behind the receiving antenna, a limiter protects the following hardware from being damaged by too high reception power, e.g., by a strong direct signal component. A quad-Vivaldi element arrangement was used as antenna. Then the received signals are first amplified using a low-noise amplifier and down-converted to an intermediate frequency using a common local oscillator for both channels. After subsequent filtering for image suppression and further amplification, the signals are sampled in the third Nyquist band at a sampling rate of 250 MHz. This sampling technique minimizes interference effects near DC.

All clock sources are synchronized over a 10 MHz reference which is generated by a GPS disciplined rubidium standard. This ensures phase coherency between the transmitter and receiver [33].

#### 2.2.2. The X-Band Transmitter Used as Illuminator

**Figure 3:**
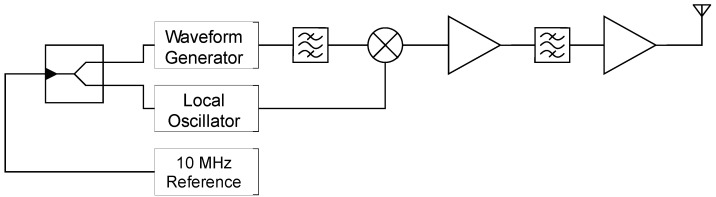
The structural schematic of the X-band transmitter used as illuminator for the bistatic radar measurements.

Figure 3 shows the transmitter of the bistatic radar setup. The transmitter is designed for the X-band and can transmit arbitrary waveforms with a bandwidth up 100 MHz and a power of 30 dBm according to the transmission regulations. A horn antenna is used as transmit antenna. The radar signals are generated by the arbitrary waveform generator (AWG) in the baseband. After up-conversion the images are filtered, and the signals are amplified. The last amplifier stage is a power amplifier directly attached to the antenna.

Both the local oscillator and the AWG digital-to-analog converter are referenced by an external 10 MHz signal and are thus synchronized with the receiver to ensure phase coherency during the measurements.

### 2.3. The Coherent Signal Processing Approach

**Figure 4:**
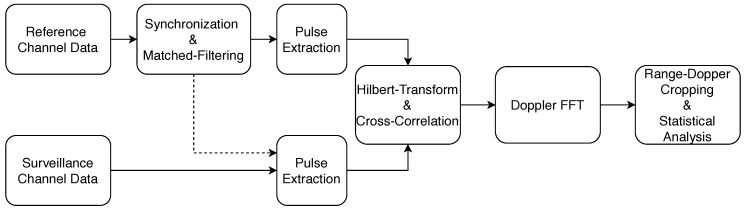
This figure shows the signal processing chain considering the two receive channels of the bistatic radar receiver. During the measurements, the reference channel antenna points to the transmitter and receives the direct signal while the surveillance channel receives the clutter echoes. The statistical parameters of the clutter for the rural terrain are calculated on the processed and cropped range-Doppler maps.

The measurement data were processed according to the coherent signal processing approach shown in Figure 4 to generate range-Doppler maps which served as a basis for the further statistical clutter amplitude analysis. A linear frequency modulated pulse waveform was used as transmit signal which is received directly by the reference channel directed to the side lobe of the transmit antenna during each measurement. Since the waveform is known, the beginning and the end of an incoming pulse are determined by a matched filter and synchronization step [34].

The corresponding samples of the incoming pulse are also extracted in the surveillance channel. The pulse pairs are then Hilbert transformed to generate complex signals. The corresponding range profiles are calculated by cross-correlating the reference and surveillance channel pulses. This calculation is determined in the frequency domain using the fast convolution. The individual range profiles are stored as columns in a matrix. The number of columns is defined by the coherent processing interval (CPI). The Doppler fast Fourier transform (FFT) is calculated along the individual lines of the matrix resulting in the range-Doppler map. The range-Doppler maps are then cropped in both domains to the dominant clutter area to perform the statistical analysis. For the measurement trials the CPI was set to 8192 pulses or correspondingly to 0.16384 s.

### 2.4. Statistical Parameters

The range-Doppler maps reduced in bistatic range and Doppler to the dominant clutter region were evaluated using various statistical parameters. Corresponding to the terrain dimensions the range-Doppler maps were cropped in bistatic range up to 500 m and in Doppler to 1.18 kHz. Accordingly, these results in a maximum number of 333 range bins and 193 Doppler bins used for the further evaluation. Only the amplitude values of the clutter patches were used for the analysis. This is due to the temperature dependence of the measuring system and the resulting phase noise between the two receiving channels. Corresponding synchronization between the receiving channels and a reliable evaluation of the phase values is part of our future work. The statistical parameters are averaged over the measurements of the respective terrain types and normalized to the corresponding mean value for better comparison because of the different damping factors caused by the terrain dimension. In the results section, histogram plots are presented to approximate the discrete probability density function (PDF) of the different terrain types. To compare the summer and winter histogram appearance of the corresponding terrain types, the Pearson correlation coefficient (PCC) is used as a measure of correlation between the different terrain types. The PCC is defined for the histograms $h_{i}$ and $h_{j}$ over several *B* histogram bins as follows:

(1) $$r_{h_{i}h_{j}}=\frac{\sum_{b=1}^{B}\left(h_{i}\left[b\right]-\bar{h_{i}}\right)(h_{j}\left[b\right]-\bar{h_{j})}}{\sqrt{\sum_{b=1}^{B}{\left(h_{i}\left[b\right]-\bar{h_{i}}\right)}^{2}}\sqrt{\sum_{b=1}^{B}{\left(h_{j}\left[b\right]-\bar{h_{j}}\right)}^{2}}}$$

where $h_{i/j}\left[b\right]$ are the corresponding histogram bins. A value of 0 is indicating no correlation between the histograms, whereas a value of 1 or −1 is indicating a positive or a negative correlation, respectively. The accumulated histograms are an indication for the cumulative distribution function (CDF). Furthermore, the skewness of the clutter amplitudes is a measure of the asymmetry of the data around the histogram mean. If the averaged skewness is less than zero the clutter amplitudes spread more to the left of the histogram mean value. For values greater than zero the spread is on the right side of the histogram mean. The skewness for the cropped range-Doppler regions is calculated as follows:

(2) $$s_{bias}=\frac{\frac{1}{MN}\sum_{m=1}^{M}\sum_{n=1}^{N}{\left(c\left[mn\right]-\bar{c}\right)}^{3}}{{\left(\sqrt{\frac{1}{MN}\sum_{m=1}^{M}\sum_{n=1}^{N}{\left(c\left[mn\right]-\bar{c}\right)}^{2}}\right)}^{3}}$$

where c[mn] is the clutter amplitude value at position m,n in the cropped range-Doppler map, $\bar{c}$ the mean amplitude value of the clutter patch, *M* the number of bistatic range bins and *N* the number of Doppler bins. Because the above calculated skewness value is biased a correction is applied according to the following formula:

(3) $$s=\frac{\sqrt{MN(MN-1)}}{MN-2}s_{bias}$$

The kurtosis is another statistical parameter quantifying the histogram appearance. The kurtosis can also be interpreted as an indicator for the dynamic range of the terrain clutter. A higher value describes a higher concentration of clutter amplitudes around a certain value and thus a lower dynamic range. The kurtosis of a distribution is defined as:

(4) $$k_{bias}=\frac{\frac{1}{MN}\sum_{m=1}^{M}\sum_{n=1}^{N}{\left(c\left[mn\right]-\bar{c}\right)}^{4}}{{\left(\frac{1}{MN}\sum_{m=1}^{M}\sum_{n=1}^{N}{\left(c\left[mn\right]-\bar{c}\right)}^{2}\right)}^{2}}$$

The calculated biased kurtosis value can be corrected as follows:

(5) $$k=\frac{MN-1}{\left(MN-2)(MN-3\right)}\left(\left(MN+1\right)k_{bias}-3\left(MN-1\right)\right)+3$$

Another parameter to quantify the clutter properties is the interquartile range (IQR). The IQR is an indicator for the dynamic range between the 75% and 25% quartile of the data neglecting the values with low probability of occurrence:

(6) $$IQR=c_{75\%}-c_{25\%}$$

where *c* is a 1×MN vector which results by reshaping the M×N clutter region. This measure of variability indicates the spread of the normalized clutter amplitude values for the different terrain types. This definition for the dynamic range of a terrain does not consider any outliers in the clutter amplitudes and is therefore suitable for a better comparison of the characteristics. A further parameter to characterize the terrain is the standard deviation of the clutter amplitudes within the reduced range-Doppler map defined as:

(7) $$\sigma =\sqrt{\frac{1}{MN-1}\sum_{m=1}^{M}\sum_{n=1}^{N}{\left(c\left[mn\right]-\bar{c}\right)}^{2}}$$

The described parameters will be used in the further course of this article for the description and comparison of the ground clutter. In the next section the results of the different measurement campaigns and the calculated parameters for the different types of terrain as well as the seasonal differences are presented.

## 3. Results

In this section, the calculated statistical parameters for the different types of German rural terrain and seasons are presented and compared. The histogram plots of the clutter amplitudes and the derived cumulative distribution functions are presented in the following Section 3.1 and Section 3.2. The descriptive statistics parameters are then listed in tabular form for each terrain group in Section 3.3.

### 3.1. Histogram Plots

**Figure 5:**
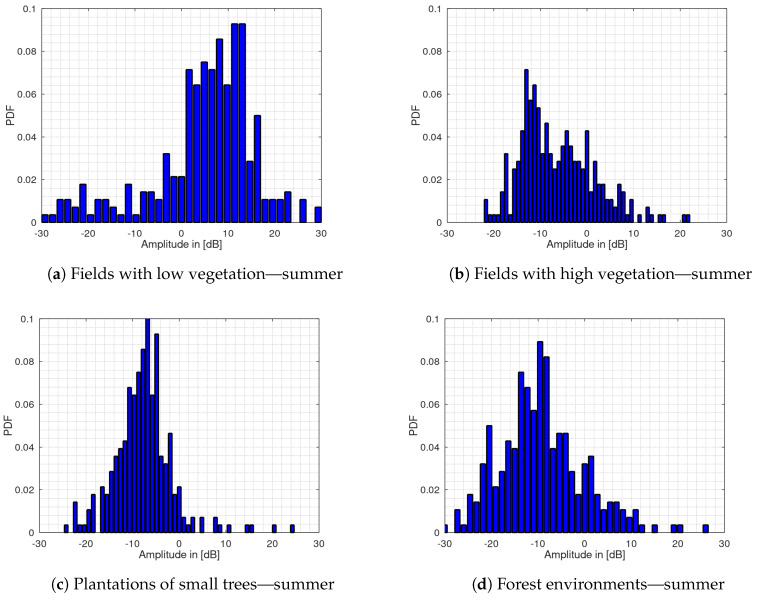
Histogram plots of the clutter amplitudes for the summer measurements for (**a**) fields with low vegetation, (**b**) fields with high vegetation, (**c**) plantations of small tress and (**d**) forest environments.

Figure 5 shows the histogram plots of the four terrain groups for the measurements carried out in the summer. The histograms are normalized to their mean values for better comparability. Otherwise the different damping factors resulting from different terrain dimensions would lead to an incorrect representation of the results. The histogram values are also normalized in their amplitude values so that they add up to one over all histogram bins. Then a cumulative sum over the bins results in the CDF. The histogram bin-range is limited to −30 dB up to 30 dB for the visualization.

It can be seen that the histogram plots show a shifted probability density function for the fields with low vegetation to the right of the mean value. For the fields with high vegetation, plantation of small trees and forests, the probability density functions are shifted to the left. It can also be seen that the PDF appearance and width are clearly different for each terrain.

**Figure 6:**
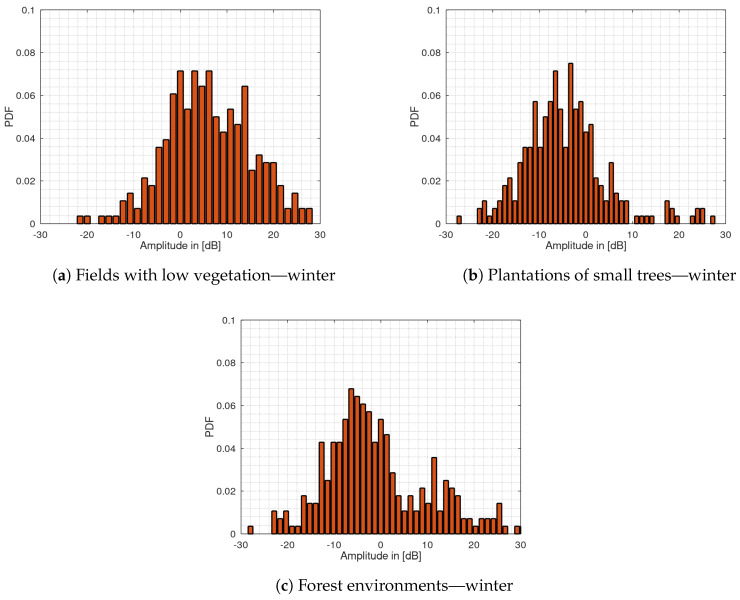
Histogram plots of the clutter amplitudes during winter measurements for (**a**) fields with low vegetation, (**b**) plantations of small trees and (**c**) forest environments.

Figure 6 shows the histogram plots for the measurements carried out in the winter. For the fields with high vegetation, no measurements have been made because during this season the terrain falls into the group of fields with low vegetation. It can also be seen in the data of the winter measurements that the PDF for fields with low vegetation also shows a shift to the right, whereas the other terrain groups show a shift to the left. This is the same behavior as for the measurements carried out during the summer. In general, the histograms for all terrain types show a flattened course for the winter measurements than the corresponding histograms for the summer measurements. The flattening of the histograms could be an indication of an increase in the clutter amplitude spread due to reduced vegetation at this time of year. To quantify the correlation of the histogram appearance for the different terrain types between summer and winter, the PCC between the fields with low vegetation, plantations of small tress and forest environments is shown in Table 1. The calculation for fields with high vegetation was not carried out, because this type of terrain is not available in winter.

As can be seen the fields with low vegetation for summer and winter correlate with a value of 0.78. Also, the histogram appearance for the plantations of small trees shows a strong correlation between summer and winter with a value of 0.76. The forest environment in summer correlates with the forest environment in winter with a value of 0.64 and a value of 0.84 with the plantations of small trees during winter. A possible explanation could be the fact that both types of terrain consist mainly of trees.

### 3.2. Clutter Cumulative Distribution Functions

**Figure 7:**
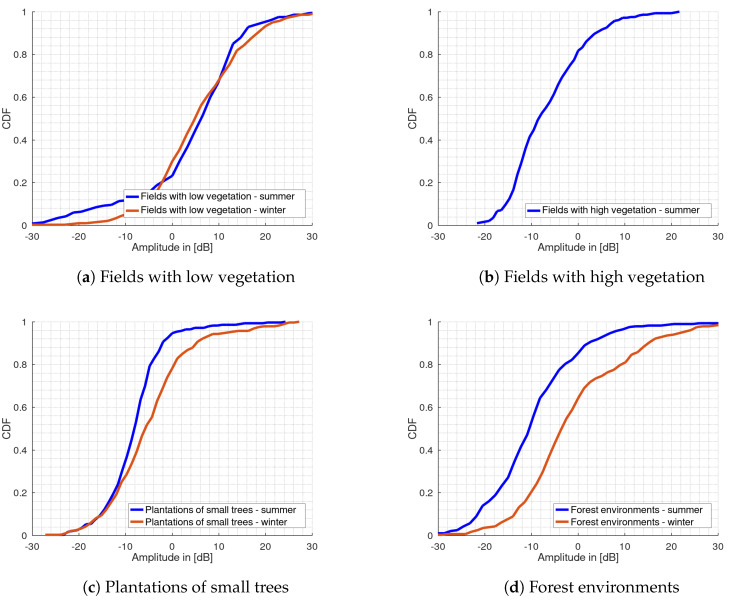
Cumulative distribution function plots of the clutter amplitudes for summer and winter for (**a**) fields with low vegetation, (**b**) fields with high vegetation, (**c**) plantations of small tress and (**d**) forest environments.

The cumulative distribution functions for the clutter amplitudes were calculated from the histogram values by cumulative summing up the histogram bins. The results are shown in Figure 7 for the various rural terrain types for winter and summer measurements. For the fields with low vegetation, only summer measurements are available in Figure 7b. As this type of terrain is only available during the summer, no measurement data were recorded. Instead, the empty fields then fall into the category of fields with low vegetation in winter. The CDF representation visualizes the density of the relative damping values. As expected, these are highest for forest environments, see Figure 7d, and lowest for fields with low vegetation in Figure 7a. During the transition from summer to winter, the density functions especially for plantations of small trees in Figure 7c and forest environments, move farther to the right. The steepest increase in slope of the CDF curve results for the terrain group of the plantations of small trees. This can also be seen from the corresponding histogram, as the amplitude values are centered around the mean value. It can be seen that the flatter the CDF curve, the larger the clutter spread.

The offset, defined as CDF shift between the forest data in summer and winter is significantly greater at about 8 dB compared to plantations of small trees with about 3 dB. For fields with low vegetation this offset is negligible. According to their CDF, these have similar statistics in both seasons. It can be further noted that the slope of the CDF is higher for the plantations than for the other types of terrain. This behavior is also confirmed in the data from the winter measurements.

### 3.3. Descriptive Clutter Statistics

Table 2 shows the characteristics of the descriptive statistics for the measured bistatic ground clutter. Here, fields with low vegetation show the largest standard deviation with nearly 12 dB. The lowest standard deviation was measured for terrains with plantations of small trees. In contrast, the data for the winter measurements give the largest IQR with about 14 dB for forest environments. As already seen in the histogram plots, the data for the fields with low vegetation were negatively skewed for both summer and winter. The highest value for the kurtosis was calculated from the measured data for the plantations of small trees during summer.

## 4. Discussion

In this article, we present the statistical analysis of bistatic radar rural ground clutter for different terrain types under grazing angles below 5°. The main novelty in this work is the separated statistical evaluation based on real measurement data of the clutter properties for different terrain subgroups forming a typical German rural environment. The selected dominant types of terrain are fields with low vegetation, fields with high vegetation, plantations of small trees and forest environments. The group of fields with low vegetation includes terrain types such as meadows or farmland. The group of fields with high vegetation includes wheat or cornfields, for example. Plantations of small trees are the group consisting of environments such as fruit tree plantations, vineyards or other arrangements of smaller trees. Forests form the last rural subgroup. Additionally, the characteristics are described for different seasons, namely summer and winter. For each terrain group, bistatic radar measurements have been carried out in the Eifel region in western Germany in the summer 2019 and winter 2019/2020 using a bistatic radar currently under development. The data from the individual measurements were processed, and the averaged PDF, CDF, standard deviation, skewness and kurtosis values were presented.

The histogram plot for the group of fields with low vegetation shows a skewness to the right both for the summer and winter measurement data. This is also indicated by the unbiased skewness value of s=−0.98 for the summer and −0.11 for the winter measurement data, whereas all other types of terrain show a positive value. The PCC shows a strong correlation between the winter and summer PDF with a value of 0.78 whereas there is no correlation with the other types of terrain. This was also verified in [20]. The kurtosis remains approximately the same with the values 1.7 and 1.3. The CDF for this terrain also shows a clear shift to the other terrain types CDF by nearly 10 dB, also again for the summer and winter, whereas the shift is less noticeable in winter. The standard deviation with σ = 11.8 dB for the summer is only reduced to 10.1 dB compared to the winter measurements while the interquartile range is increased by nearly 3 dB. This behavior contrasts with the other types of terrain where the standard deviation generally increases in winter, whereas the IQR increases also for all other types of terrain. For the other groups, these changes are greater. However, bistatic measurements in such environments with low vegetation suffer from a strong direct signal component arriving at both receive channels. A reduction in detection performance is then probably mainly due to the direct signal instead of the clutter caused by the low vegetation. Since the current setup does not have an automatic gain control an optimal dynamic range of the analog-to-digital converter is not guaranteed. For this reason, the validity of the determined parameters for this terrain cannot be confirmed and thus may explain the behavior of the standard deviation. Corresponding work on the hardware is part of our future work.

The histogram plots for the remaining environments are all shifted to the left around the histogram mean value also both for summer and winter measurement data while the shift slightly decreases for the winter measurements. The correlation analysis shows that the histograms of plantations and forests are strongly correlated, which may be explained by the fact that both types of terrain consist mainly of trees. With a value of 0.98, the skewness is much more dominant for the plantations of small trees in the summer compared to the other terrain types. This is also the case for the kurtosis with a value of 4.4. The values for skewness and kurtosis are the highest in the data compared to the other types of terrain. This indicates a lower dynamic range because the amplitude values are very concentrated which may be due to the nature of this vegetation, which consists mainly of small leaves with high water content and dense vegetation with branches. The standard deviation and the IQR show the lowest value with 6.2 dB and 6.3 dB respectively for both summer and winter measurements. Since both values can be regarded as an indicator for the dynamic range, it can be seen that the plantations lead to significant signal attenuation and less dynamic range, especially in the summer when the trees are covered with water-filled leaves or fruit.

For the fields with high vegetation, the standard deviation shows a similar behavior as for the plantations with only a difference of 1.7 dB. However, the IQR of 11.2 dB is significantly higher and thus as high as for forest environments. Together with a value of 0.5 for the kurtosis, this indicates a high dynamic range of the clutter values. This can also be seen by the flattened histogram plot. A possible cause could be movements of the vegetation itself e.g., grain plants in the wind, resulting in a greater amplitude spread. The dynamic range for the forest environments is also with a value of 11.1 dB in the summer and 14.4 dB in the winter significantly higher compared to the other terrain types. It can be noted, although the plantations and forests consist mainly of trees, they differ significantly in the dynamic range of the clutter. A possible explanation could be that the plantations have a much denser vegetation and thus less multipath propagation are formed.

As aforementioned for all terrain types, except for the fields with low vegetation, an increase in the standard deviation from the summer to the winter statistics is visible and a respective shift of their CDFs. For plantations, the increase in the standard deviation is greatest with a value of 2.8 dB. This could be due the fact that the plantation trees are covered with many smaller, thicker leaves in summer, while these are missing in winter. As a result, considerably more propagation paths are formed which could lead to this higher value. This effect can also be seen with IQR value that has increased by almost 4 dB compared to the summer measurements. The clutter amplitudes for the forest environments also show the same behavior, although the increase in standard deviation and IQR of about 1.7 dB and 3 dB is lower, but the total values are still higher. Again, the low number of leaves in winter is a possible explanation for the increase. For both types of terrain, this behavior can also be seen from their CDF which shows the largest differences between the summer and winter data. Similar results were achieved for forest environments in [13].

For all types of terrain, there is a decrease in kurtosis values from summer to winter. This decrease is again particularly strong for forests and plantations of small trees with 4.4 and 2.3 in the summer compared to 1.64 and 0.33 in the winter, respectively. However, this result is in line with the increase of the standard deviation and the increase of the IQR. As the clutter amplitudes are distributed over a larger amplitude range in the data for the winter measurements, the histograms must be flattened which leads to a reduced kurtosis.

Interestingly, all terrain types, except the fields with low vegetation show a shift of their CDF to the right in the winter compared to the data collected in summer. As mentioned, the fields with low vegetation, however, have the largest offset of their CDF with more than 10 dB compared to other measurements but show a similar density function for summer and winter. This could be because the differences in vegetation between summer and winter is very small for fields with low vegetation, whereas all other types of terrain show significant differences in vegetation.

However, it must be noted that in this survey the different types of terrain were only measured at an angle of 45 degrees between transmit and reference antenna boresight. More reliable statements can only be made by further measurements at different aspect angles. This circumstance contrasts with a not inconsiderable measurement effort. In our future work we plan to place the bistatic receiver on a moving platform [35] to cover a wide range of aspect angles by driving along with the different terrain types if possible due to their accessibility. A similar measuring principle is known from the field of antenna measurement, where any spatial points can be measured.

It should be mentioned that another important field of application for the processed statistical parameters is in the area of knowledge-aided constant false alarm rate (CFAR) [36]. By knowing the clutter statistics of the rural terrain, the threshold value for the target detection can be adapted to the scene and thus may leading to an increase in the detection performance. This can also be supported by a classification of the clutter PDF’s based on the corresponding PCC values. This is currently the subject of our ongoing research work.

## 5. Conclusions

This article presents the statistical analysis of amplitude properties from bistatic radar ground clutter for rural environments country-specific to Germany. Therefore, the rural environment was divided into relevant subgroups, namely fields with low vegetation, fields with high vegetation, plantations of small trees and forest environments. To investigate the influence of the seasons on the clutter properties, bistatic radar measurements were carried out for low grazing angles in summer and winter in the Eifel region in western Germany. Range-Doppler maps were created from the sampled data, and the statistical properties were determined on a cropped region of the maps covering the dominant clutter. It has been shown that all types of terrain differ considerably in their statistical parameters and therefore a separate consideration is justified and necessary. For the terrain group of fields with low vegetation, there is no significant difference between the PDF and CDF for summer and winter in amplitude characteristics also indicated by the presented correlation analysis, but an increase in dynamic range of 2.1 dB. In general, the measured data for this type of terrain showed a negative skewness with −0.98 and −0.11 for both seasons, whereas the other terrains indicate positive values. This may be caused by the strong direct signal present during the measurements where an automatic gain control in our future work may reduce this effect. Plantations with small trees, on the other hand, show an increase of the standard deviation and the IQR of 2.8 dB and 3.8 dB respectively in winter compared to summer. Their statistical properties differ significantly from those of fields with low and high vegetation, which is for example shown by a kurtosis value of 4.4 compared to 1.7 and 0.5. The clutter dynamic range in terms of IQR for the forest environments is in general higher with 11.1 dB in the summer and 14.4 dB in the winter measurements compared to the plantations with 6.3 dB and 10.1 dB may be due to the much denser vegetation of the latter. Especially the differences between summer and winter are more pronounced compared to fields with low vegetation with only 2.3 dB. This could be due to the lack of leaves in winter and an increase of multipath propagation whereas fields with low vegetation are not drastically changing their appearance in the winter. For our future work, further measurement campaigns are planned, especially with a moving receiver. This will further verify the statistical parameters and allow many aspect angles to be measured in a single measurement. By extending our hardware we will also be able to evaluate the clutter phase response. Nevertheless, the data presented are an important input for adaptive signal processing and adaptive signal detection, respectively. Among other things, work in the field of knowledge-aided CFAR is planned to generate an increase in detection performance by means of an adapted detection threshold to the clutter environment. The results obtained are also of great interest for future applications in the field of adaptive detection, such as knowledge-aided space-time adaptive processing (STAP), since the clutter signal space can be modeled accordingly, rather than assuming homogeneous environments [37]. This also includes general detectors based on hypothesis testing, for example, since the disturbance in the form of the environmental clutter can be better modeled by such statistical models and thus leading to an increase in detection performance [38].

## Figures and Tables

**Table 1:** Pearson correlation coefficients for the different clutter histograms of the fields with low vegetation, plantations of small trees and forest environments and the corresponding winter histograms.

Terrain under Test	Fields with Low Vegetation Winter	Plantations of Small Trees Winter	Forest Environment Winter
Fields with low vegetation summer	0.78	0.1	0.18
		
Plantations of small trees summer	−0.16	0.76	0.59
		
Forest environment summer	−0.04	0.84	0.64
		

**Table 2:** Statistical properties of the clutter amplitudes for the different terrain types during summer and winter.

Terrain under Test	σ	IQR	Skewness	Kurtosis
Fields with low vegetation—summer	11.8 dB	10.8 dB	−0.98	1.7
Fields with high vegetation—summer	7.9 dB	11.2 dB	0.73	0.5
Plantations of small trees—summer	6.2 dB	6.3 dB	0.98	4.4
Forest environment—summer	10.0 dB	11.1 dB	0.88	2.3
Fields with low vegetation—winter	10.1 dB	13.1 dB	−0.11	1.3
Plantations of small trees—winter	9.0 dB	10.1 dB	0.84	1.64
Forest environment—winter	11.7 dB	14.4 dB	0.62	0.33

## Abbreviations

The following abbreviations are used in this manuscript:

PCL	Passive Coherent Location
SNIR	Signal-To-Interference-Plus-Noise Ratio
AWG	Arbitrary Waveform Generator
CPI	Coherent Processing Interval
FFT	Fast Fourier Transform
PDF	Probability Density Function
PCC	Pearson Correlation Coefficient
CDF	Cumulative Distribution Function
IQR	Interquartile Range
CFAR	Constant False Alarm Rate
STAP	Space-Time Adaptive Processing
